# Radiological Parameters for the Detection of Pulmonary Hypertension in Severe Aortic Valve Stenosis and Their Influence on Mortality: Does Sex Matter?

**DOI:** 10.3390/jcm13071999

**Published:** 2024-03-29

**Authors:** Joseph Kletzer, Bernhard Scharinger, Ozan Demirel, Reinhard Kaufmann, Michaela Medved, Christian Reiter, Matthias Hammerer, Clemens Steinwender, Stefan Hecht, Kristen Kopp, Uta C. Hoppe, Klaus Hergan, Elke Boxhammer

**Affiliations:** 1 Department of Internal Medicine II, Division of Cardiology, Paracelsus Medical University of Salzburg, 5020 Salzburg, Austria; 2 Department of Radiology, Paracelsus Medical University of Salzburg, 5020 Salzburg, Austria; 3 Department of Cardiology, Kepler University Hospital, Medical Faculty of the Johannes Kepler University Linz, 4040 Linz, Austria

**Keywords:** aortic valve stenosis, computed tomography angiography, pulmonary hypertension, PA diameter, PA/BSA, TAVR

## Abstract

**Background:** Echocardiography has long been established as the primary noninvasive method for diagnosing pulmonary hypertension (PH) prior to transcatheter aortic valve replacement (TAVR) in patients with severe aortic valve stenosis (AS). In recent years, radiological methods for diagnosing PH have been investigated. Measurements such as the computed tomography angiography (CTA)-derived pulmonary artery (PA) diameter and PA diameter/body surface area (PA/BSA) have shown promising results regarding their diagnostic strength. However, it has yet to be determined if a patient’s sex has any impact on the effectiveness of these diagnostic measurements. **Methods:** In all, 271 patients (51.3% male, mean age 82.6 ± 4.8 years) with severe AS undergoing TAVR were separated into male and female groups. The cut-off values for the diagnosis of PH were calculated for the CTA-derived PA diameter and PA/BSA based on different systolic pulmonal artery pressure values (40–45–50 mmHg). Patients were then subclassified according to measurements above or below these PA diameters and PA/BSA cut-off values. A PA diameter ≥29.5 mm and PA/BSA ≥ 15.7 mm/m^2^ qualified for PH. The 1–5 year survival rate in these cohorts was further analyzed. **Results:** Patients with a PA diameter ≥29.5 mm showed a significantly higher 1 year mortality rate (*p* = 0.014). This observation could only be confirmed for the male sex (*p* = 0.018) and not for the female sex (*p* = 0.492). As for the PA/BSA, in patients over the cut-off value, no significant increase in mortality was noted in the overall cohort. However, the male patients showed increased 3 year (*p* = 0.048) and 5 year mortality rates (*p* = 0.033). **Conclusions:** The CTA-obtained PA diameter and PA/BSA are both useful in the diagnosis of PH and mortality risk stratification in patients with severe AS undergoing TAVR, especially in males. Male patients with PA ≥ 29.5 mm or PA/BSA ≥ 15.7 mm/m^2^ seem to be at a higher risk of death during follow-up after undergoing TAVR. In females, no such correlation was observed.

## 1. Introduction

Pulmonary hypertension (PH) is a common concomitant disease in patients with severe aortic valve stenosis (AS) [1,2]. It has been shown to decrease survival and increase the complication rates in surgical as well as interventional repair of AS [3,4]. Therefore, diagnosis of PH prior to transcatheter aortic valve replacement (TAVR) is pivotal. In recent years, right heart catheterization to assess pulmonary pressure for the diagnosis of PH has been largely replaced by less-invasive methods such as echocardiographic measurements of systolic pulmonary artery pressure (sPAP) [5]. According to the currently available literature, sPAP lends itself as an accurate diagnostic measurement, as well as an independent risk factor for mortality [6,7]. Definitive cut-off values for sPAP remain unclear due to a lack of consensus in this regard. Currently, sPAP cut-off values ranging from ≥40 mmHg to ≥50 mmHg have been proposed in various studies and guidelines [3,4,6,8,9].

Since computed tomography angiography (CTA) is strongly recommended during the workup preceding TAVR intervention, and as current European Society of Cardiology (ESC) guidelines also suggest CTA imaging as an alternative for noninvasive PH diagnosis [10], our working group recently investigated CTA-derived measurements such as the pulmonary artery (PA) diameter and PA diameter/body surface area (PA/BSA) and their potential in the diagnosis of PH prior to TAVR [8,11,12]. Regarding the PA/BSA measurements, no statistically significant differences were found in the mortality rates of patients above and below the calculated cut-off values [8]. Considering that sex plays a substantial role in all aspects of cardiovascular disease [13], this study aims to investigate the influence of sex on the diagnostic strength of CTA measurements for PH such as the PA diameter and PA/BSA and their potential for mortality prediction in patients with severe AS undergoing TAVR.

## 2. Materials and Methods

### 2.1. Study Population

In total, 271 patients (51.3% male, mean age 82.6 ± 4.8 years) with severe, primary degenerative AS undergoing TAVR procedures between 2016 and 2018 were analyzed retrospectively. The exclusion criteria for this study were the presence of a bicuspid aortic valve, acute cardiac decompensation at the time of transthoracic echocardiography or at the time of TAVR, as well as evidence of the presence of pre-capillary PH (chronic thromboembolic PH, idiopathic PH, interstitial lung disease or underlying rheumatologic disease with pulmonary involvement, etc.). The study protocol was approved by the local ethics committees of the Paracelsus Medical University Salzburg (415-E/1969/5-2016) and Johannes Kepler University Linz (E-41-16). Consent was obtained from all patients in written form.

### 2.2. Transthoracic Echocardiography

The ESC guidelines were used to classify AS, and valve disease was categorized as severe when the AV Vmax (the maximum velocity across the aortic valve) was 4.0 m/s, AV dpmean (mean pressure gradient across the aortic valve) was ≥40 mmHg, and aortic valve area (AVA) was ≤1.0 cm^2^. Mitral, aortic, and tricuspid valve regurgitation, classified as minimal, mild, moderate, and severe, were diagnosed using spectral and color Doppler images. For calculation of the left ventricular ejection fraction (LVEF), Simpson’s method was used. To calculate the sPAP, the tricuspid regurgitant jet velocity (TRV), and right atrial pressure (RAP) were needed. The TRV was recorded using continuous wave Doppler over the tricuspid valve. The estimated RAP was obtained by determining the inferior vena cava (IVC) diameter, as it corresponds to the central venous pressure. Details for the RAP estimation can be found in Table 1. To calculate the sPAP, we used the simplified Bernoulli equation (4 × TRV2) + RAP. Common echocardiographic devices (iE33 and Epiq 5; Phillips Healthcare, Hamburg, Germany) were used during examination.

### 2.3. CTA Protocol and PA Diameter Measurement

**Figure 1:**
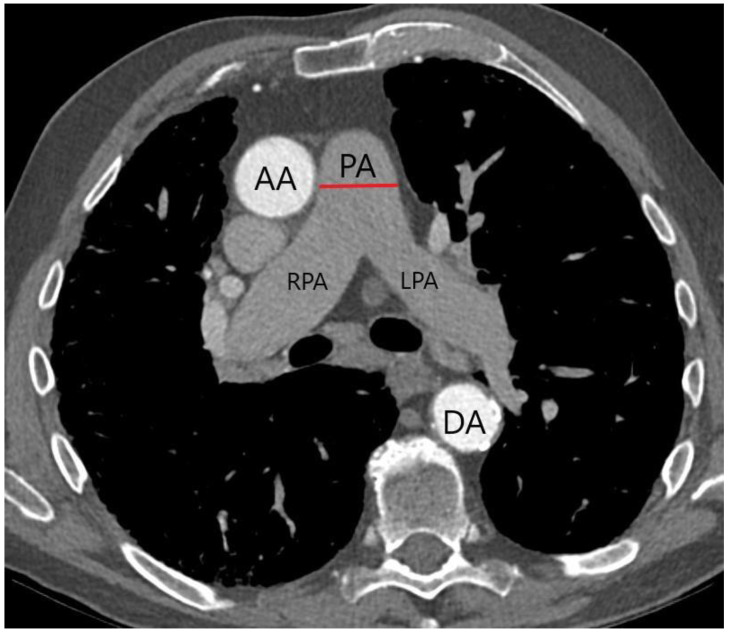
Measurement of PA diameter (red line) on axial CTA. PA = pulmonary artery; RPA = right pulmonary artery; LPA = left pulmonary artery; AA = ascending aorta; DA = descending aorta.

To ensure optimal preparation, each patient received a CTA workup before TAVR. This included evaluation of the aortic annulus size, aortic anatomy, and femoral vascular access. Examinations were performed using electrocardiogram (ECG)-triggered CT-angiography (second-generation, multidetector 256 or 128 slice dual source CT (Revolution, General Electric Healthcare, IL, USA) or Somatom Definition AS+ (Siemens Healthcare, Erlangen, Germany)). Non-ionic iodinated contrast media were infused as a 100 mL bolus followed by 70 mL 0.9% saline solution at a rate of 3.5–5 mL/s. CT images were acquired in an early arterial phase and then assessed by two separate radiologists who did not have any previous knowledge of the patients’ clinical information. The PA diameter was measured as can be seen in Figure 1. Mean values were established between investigator 1 and 2 in each case. The BSA was calculated utilizing the DuBois formula (BSA = 0.007184 × Height0.725 × Weight0.425). All radiological analyses were performed on stationary workstations (Impax, Agfa-Gevaert, Mortsel, Belgium).

### 2.4. TAVR

The decision to pursue TAVR was made collaboratively by a diverse team of medical professionals, including cardiologists and cardiac surgeons. Specifically, all 271 patients underwent TAVR using a transfemoral approach and a CoreValve prosthesis (Medtronic, Dublin, Ireland).

### 2.5. Statistical Analysis

A sample size calculation was conducted using G*Power 3.1. We employed a *t*-test from the means test family, performed an a priori power analysis, and set specific parameters. These included an effect size (d) of 0.5, an alpha error of 0.05, a power (1 minus beta error) of 0.95, and an allocation ratio of 1. This analysis suggested that the ideal sample size for each gender group in this study should have been 105 patients. However, with a current sample size of over 130 patients per gender, our study achieved a robust power level of 0.98 given the parameters mentioned. Further data were analyzed using IBM SPSS statistics 25 (Armonk, NY, United States). A test of normality was performed using the Kolmogorov–Smirnov test and Q-Q plots for visual assessment. Data were expressed as mean ± standard deviation (SD) if they were metric and normally distributed. For the remaining metric, non-normally distributed data were stated as the median ± interquartile range (IQR). Non-metric data were conveyed as absolute and percentage values. The Student’s *t*-test was used to compare the means of normally distributed data, while the Mann–Whitney U test was used for non-parametric data. The χ2-Test was used for categorical data. The cut-off values of the PA diameter measurements and PA/BSA values in male and female patients were calculated using area under receiver operating characteristic (AUROC) curves with area under the curve (AUC) and Youden index (YI) analyses. These were performed with respect to different sPAP values, namely 40 mmHg, 45 mmHg, and 50 mmHg. The PA diameter cut-off for the diagnosis of PH used in this study was determined to be a PA diameter ≥29.5 mm. The PA/BSA cut-off was PA/BSA ≥ 15.7 mm/m^2^. Kaplan–Meier curves and log-rank tests were performed to compare survival in male and female patients with PA diameters and PA/BSA values below and above the cut-off values stated before. Univariable cox regression in regard to the 1 year, 3 year, and 5 year mortality rates in males and females was performed for each baseline characteristic mentioned in Table 2. All variables with a *p* value < 0.100 were included in a subsequent multivariable cox regression analysis. The threshold for statistical significance was set at *p* ≤ 0.05.

## 3. Results

### 3.1. Baseline Characteristics

No significant difference was noted regarding the sex distribution in our study. As expected, significant height differences (172.8 cm ± 6.6 cm vs. 160.4 cm ± 6.0 cm; *p* < 0.001) and weight differences (77.7 kg ± 12.9 kg vs. 69.3 kg ± 15.2 kg; *p* < 0.001) were present between the male and female patients. There was a significantly higher number of male patients with BMIs of 25.0–29.9 (40.4% vs. 23.5%; *p* = 0.008), whereas the female patients showed higher numbers of BMIs between 30.0 and 34.9 (10.3% vs. 18.2%; *p* = 0.039). Moreover, atrial fibrillation was also significantly more prevalent in the male patients included in our study (41% vs. 25.8%; *p* = 0.008). Male participants also showed higher numbers of pacemaker implantation after TAVR (30.2% vs. 15.2%; *p* = 0.003). The STS score was significantly higher in female patients (2.0 ± 1.2 vs. 3.5 ± 2.6; *p* < 0.001). In general, biomarkers showing statistically significant differences were higher in males (Table 2).

There was no statistical difference in mean PA diameter between males and females (29.4 mm ± 5.3 mm vs. 29.1 mm ± 4.9 mm; *p* = 0.552), nor was there a difference in the number of patients over the predetermined cut-off values (48.9% vs. 44.7%; *p* = 0.486). However, we observed a significant difference in the PA/BSA ratio in females compared with males (15.4 mm/m^2^ ± 2.7 mm/m^2^ vs. 17.0 mm/m^2^ ± 3.0 mm/m^2^; *p* < 0.001). The percentage of patients over the cut-off value was also significant (42.6% vs. 69.4%; *p* < 0.001). No significant difference was noted when comparing sPAP values. All collected baseline characteristics can be found in detail in Table 2.

### 3.2. AUROC: sPAP vs. PA Diameter

**Figure 2:**
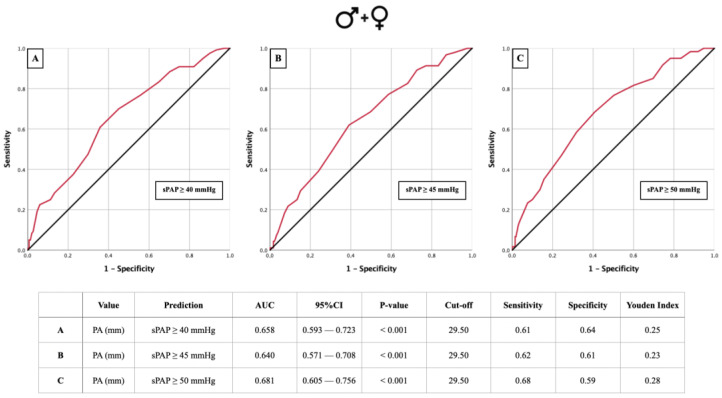
AUROC curves and tabular overview of PA diameter values of the overall cohort (**A**–**C**) for the prediction of sPAP ≥ 40, 45, and 50 mmHg with cut-off values, sensitivity, specificity, and Youden indexes. PA = pulmonary artery; sPAP = systolic pulmonary artery pressure; AUC = area under the curve; CI = confidence interval.

**Figure 3:**
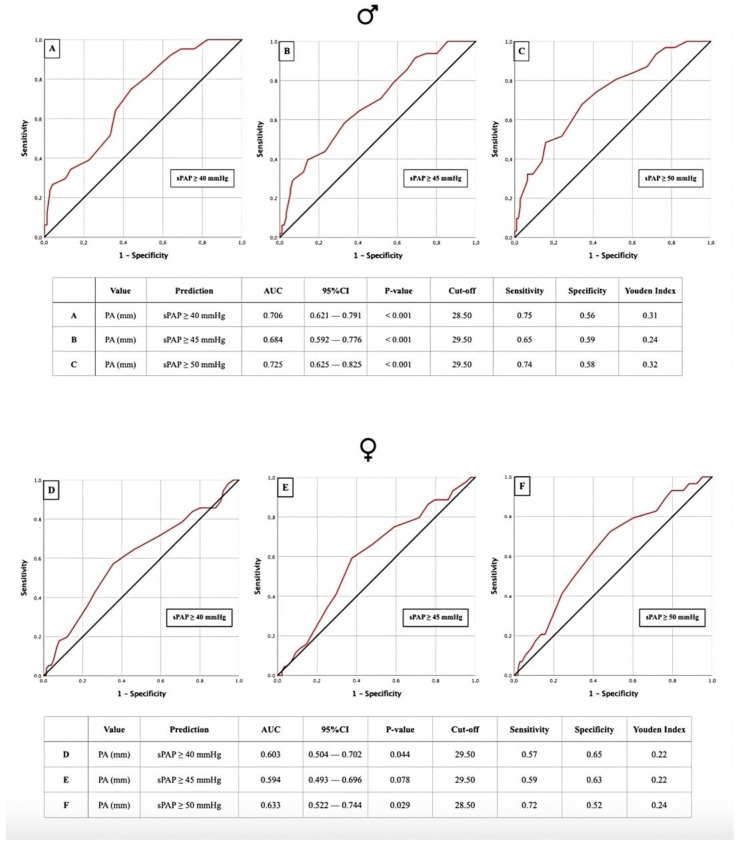
AUROC curves and tabular overview of PA diameter values of male patients (**A**–**C**) and female patients (**D**–**F**) for the prediction of sPAP ≥ 40, 45, and 50 mmHg with cut-off values, sensitivity, specificity, and Youden indexes. PA = pulmonary artery; sPAP = systolic pulmonary artery pressure; AUC = area under the curve; CI = confidence interval.

AUROC analyses for the PA diameter regarding three different sPAP cut-offs were performed to diagnose PH in the overall study cohort (Figure 2A–C), as well as in male and female patients separately (Figure 3A–F). For all sPAP cut-offs in the total cohort, a PA diameter cut-off value of 29.5 mm was calculated (Figure 2A–C). For sPAP ≥ 40 mmHg in the male cohort, we determined the PA diameter cut-off to be 28.5 mm. A cut-off of 29.5 mm was calculated for sPAP ≥ 45 mmHg and sPAP ≥ 50 mmHg (Figure 3A–C). For the females, a PA diameter cut-off of 29.5 mm was calculated for sPAP ≥ 40 mmHg. For sPAP ≥ 50 mmHg, the PA cut-off was 28.5 mm (Figure 3D–F). All calculations resulted in statistically significant cut-off values, except for sPAP ≥ 45 mmHg in females.

### 3.3. AUROC: sPAP vs. PA/BSA Ratio

**Figure 4:**
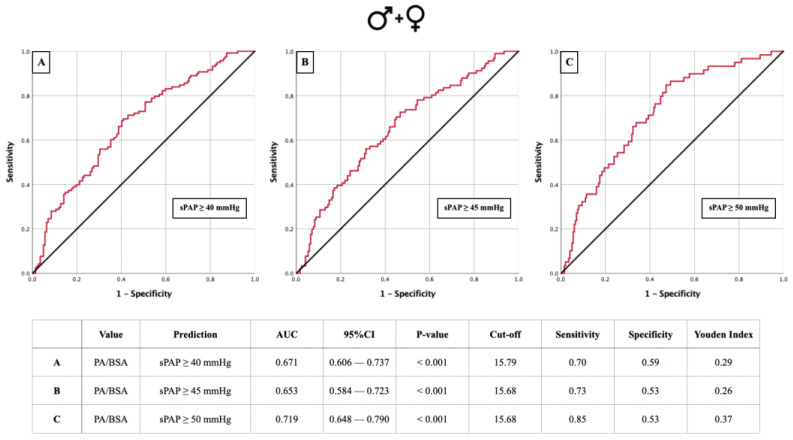
AUROC curves and tabular overview of PA/BSA values of the overall cohort (**A**–**C**) for the prediction of sPAP ≥ 40, 45, and 50 mmHg with cut-off values, sensitivity, specificity, and Youden indexes. PA = pulmonary artery; BSA = body surface area; sPAP = systolic pulmonary artery pressure; AUC = area under the curve; CI = confidence interval.

**Figure 5:**
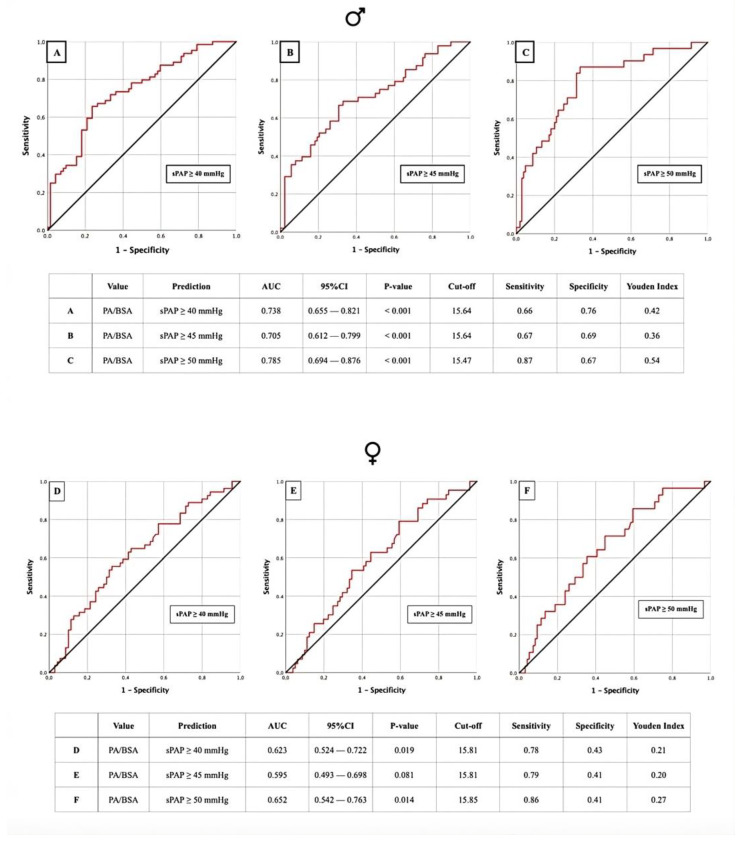
AUROC curves and tabular overview of PA/BSA values of male patients (**A**–**C**) and female patients (**D**–**F**) for the prediction of sPAP ≥ 40, 45, and 50 mmHg with cut-off values, sensitivity, specificity, and Youden indexes. PA = pulmonary artery; BSA = body surface area; sPAP = systolic pulmonary artery pressure; AUC = area under the curve; CI = confidence interval.

AUROC analyses to calculate the PA/BSA cut-offs were also performed for the same sPAP values (Figure 4 and Figure 5). In the total cohort, a PA/BSA cut-off of 15.79 mm/m^2^ was determined for sPAP ≥ 45 mmHg. Here, sPAP ≥ 40 mmHg and ≥50 mmHg had the same PA/BSA cut-off of 15.68 mm/m^2^ (Figure 4A–C). In males, the PA/BSA cut-off for sPAP ≥ 40 mmHg and sPAP ≥ 45 mmHg was determined to be 15.64 mm/m^2^. For sPAP ≥ 50 mmHg, it was 15.47 mm/m^2^ (Figure 5A–C). Lastly, in females, a PA/BSA cut-off of 15.81 mm/m^2^ was calculated for sPAP ≥ 40 mmHg and ≥45 mmHg, and sPAP ≥ 50 mmHg resulted in a cut-off of 15.85 mm/m^2^ (Figure 5D–F). All calculations resulted in statistically significant cut-off values, except for sPAP ≥ 45 mmHg in females. 

### 3.4. Cox Regression for Mortality

Table 3 shows the results for the cox regression mortality analyses for the male patients. In the multivariable regression analysis for 1 year mortality, fatal stroke after TAVR (*p* = 0.002), and sPAP (*p* = 0.004) were statistically significant. After 3 years, only sPAP was statistically significant (*p* = 0.026). The PA diameter was barely not significant (*p* = 0.052). Regarding 5 year mortality, only the PA diameter was statistically significant (*p* = 0.014). The results for the cox regression analyses of mortality in female patients can be found in Table 4. The creatinine kinase (CK) levels were statistically significant for the prediction of 1 year mortality (*p* < 0.001), 3 year mortality (*p* = 0.048), and 5 year mortality rates (*p* = 0.001) in female patients. Stroke after TAVR was highly significant as a predictor of mortality after 5 years (*p* < 0.001). No other predictor was statistically significant for the timeframes mentioned.

### 3.5. Kaplan–Meier Survival Analysis: PA Diameter- and PA/BSA Cut-off

**Figure 6:**
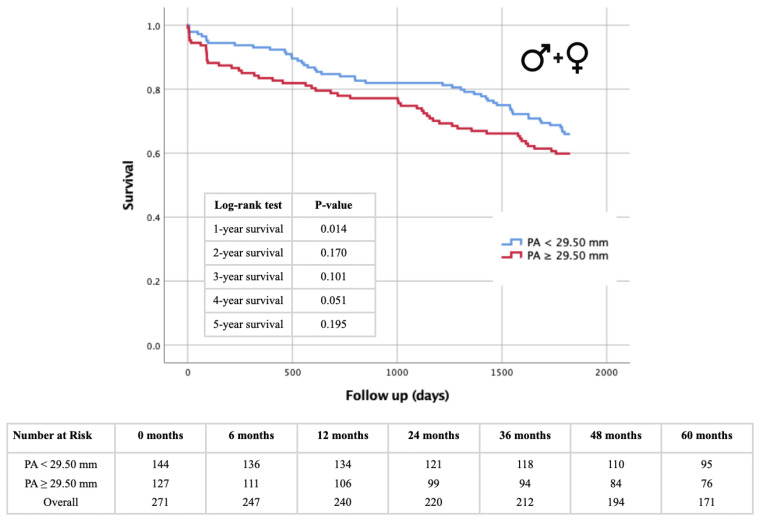
Kaplan–Meier curves for 1–5 year survival rates depending on PA diameter cut-off value ≥ 29.5 mm and table of patients included in different follow-up periods for the overall cohort. PA = pulmonary artery.

**Figure 7:**
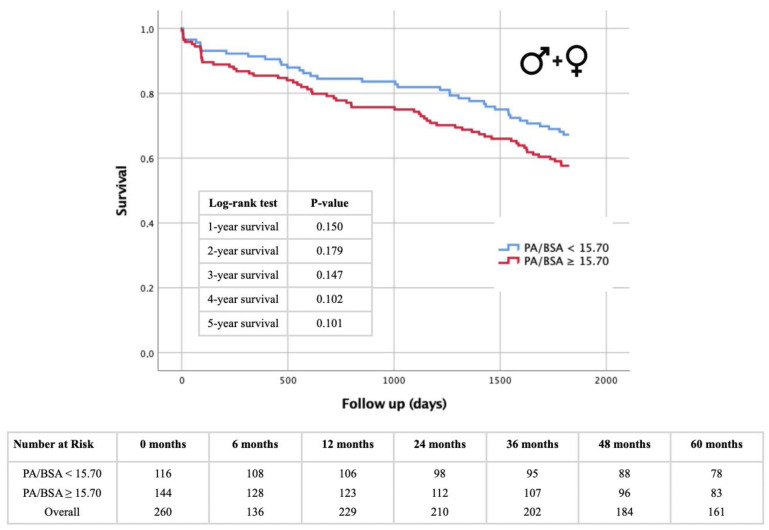
Kaplan–Meier curves for 1–5 year survival rates depending on PA/BSA cut-off value ≥15.7 mm/m2 and table of patients included in different follow-up periods for the overall cohort. PA = pulmonary artery; BSA = body surface area.

**Figure 8:**
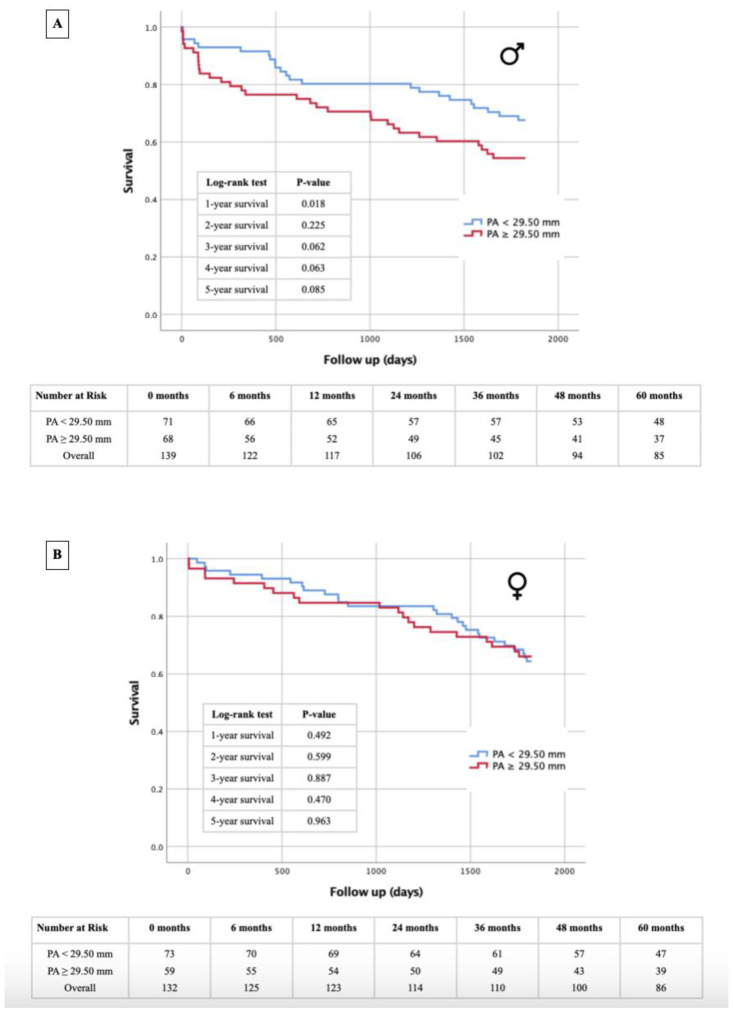
Kaplan–Meier curves for 1–5 year survival rates depending on PA diameter cut-off value ≥29.5 mm and table of patients included in different follow-up periods for male patients (**A**) and female patients (**B**). PA = pulmonary artery.

**Figure 9:**
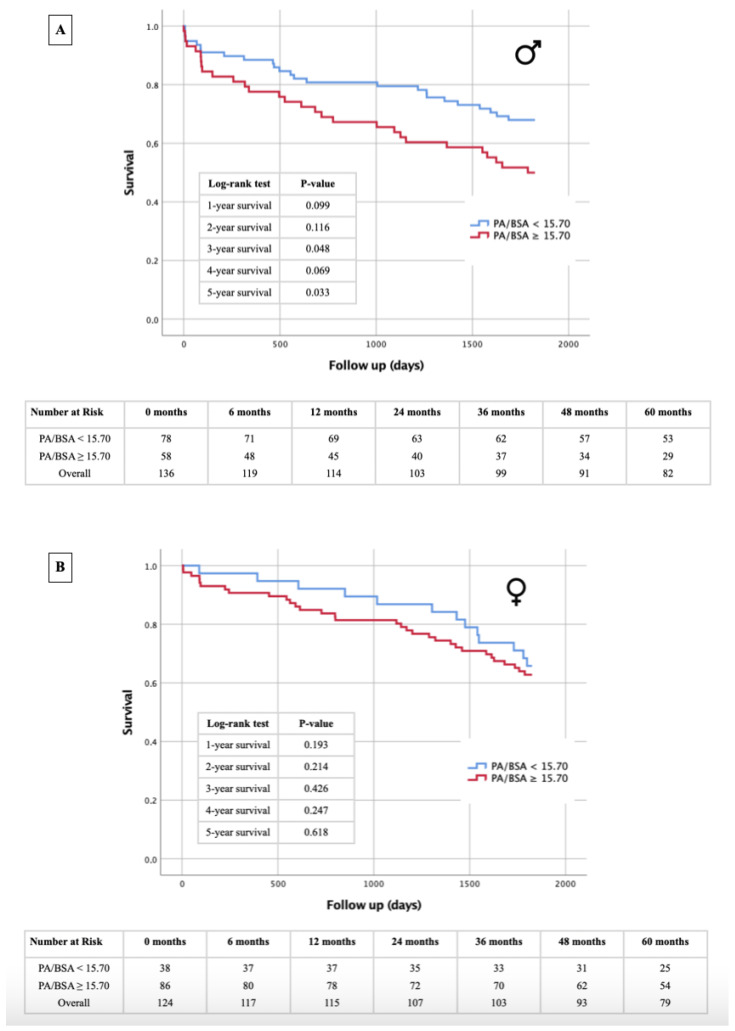
Kaplan–Meier curves for 1–5 year survival depending on PA/BSA cut-off value ≥15.7 mm/m^2^ and table of patients included in different follow-up periods for male patients (**A**) and female patients (**B**). PA = pulmonary artery; BSA = body surface area.

Figure 6, Figure 7, Figure 8 and Figure 9 illustrate the results of the Kaplan–Meier survival analyses using a PA diameter cut-off of ≥29.5 mm and a PA/BSA cut-off of 15.7 mm/m^2^. In the total cohort, using the PA diameter cut-off, the 1 year survival rate showed a statistically significant difference in the log-rank test (*p* = 0.014), and statistical significance was just missed at the 4 year survival time point, with *p* = 0.051 (Figure 6). The PA/BSA cut-off did not show a statistical significance in terms of survival rate (Figure 7). The PA diameter cut-off in male patients showed statistical significance after 1 year (*p* = 0.018). The 3 year (*p* = 0.062) and 4 year survival rates (*p* = 0.063) reached borderline *p* values but were still not statistically significant (Figure 8A). Using the PA/BSA cut-off, the 3 year survival (*p* = 0.048) and 5 year survival rates (*p* = 0.033) were both statistically significant using the log-rank test (Figure 9A). In females, there was no statistical significance noted (Figure 8B and Figure 9B).

## 4. Discussion

The main aim of this study was to compare the prognostic and diagnostic PH values of the PA diameter and PA/BSA ratio in males and females with severe AS undergoing TAVR. Currently, there seems to be limited research investigating sex differences in these relationships.

### 4.1. PA and PA/BSA: Overall Differences with Other Studies

The PA diameter cut-off of 29.5 mm for the overall cohort was calculated using AU-ROC analysis at different sPAP values (40–45–50 mmHg). Similar cut-off values were also reported by the available literature. For example, in the study of Kalinczuk et al., a cut-off of 29.3 mm was used. The authors determined that PA diameters over the cut-off were an independent predictor of 1 year mortality in the overall cohort [14]. Similarly, another study by Koseki et al. found that a PA diameter ≥29 mm was associated with 2 year all-cause mortality compared with patients below this cut-off. Additionally, they also suggested that the PA diameter might be linked to mortality, with an adjusted hazard ratio of 2.21 (*p* < 0.001) [15]. A meta-analysis from 2014 found that the cut-off value might lie between 25 mm and 33.5 mm [16]. In our work, we found that PA diameters ≥29.5 mm were associated with increased 1 year mortality (*p* = 0.014). Additionally, although not statistically significant, the results for the 4 year mortality rate came exceedingly close to but did not reach the threshold of significance (*p* = 0.051). Regarding the diagnostic value of the PA diameter for PH, when using sPAP as a reference, we arrived at higher AUC values (AUC = 0.658–0.681) compared with a previous study by Rehman et al. (AUC = 0.591) [17]. The PA/BSA cut-off of 15.7 mm/m^2^ was determined in the same fashion. When looking at the literature, Sudo et al. arrived at a value of 16.8 mm/m^2^ [18]. Additionally, in our previous work investigating the utility of the PA/BSA ratio in patients with severe AS undergoing TAVR, a PA/BSA cut-off of 16.6 mm/m^2^ was calculated [8]. Between those studies, only Sudo et al. found a significantly higher 2 year mortality rate in patients with large PA/BSA values [18]. In both this study and our previous work, no significant difference in mortality for patients above or below the PA/BSA cut-off value was found when looking at the overall patient cohort [8]. However, when taking a closer look at the PA/BSA values in our study, the female sex especially showed significantly higher values (17.0 ± 3.0 mm/m^2^ vs. 15.4 ± 2.7 mm/m^2^) as well as a higher percentage of patients with a PH cut-off value ≥15.70 mm/m^2^ (69.4% vs. 42.6%), with generally better survival. As women in most cases have a lower BSA, a higher PA/BSA ratio for the female sex would therefore be expected. This sex-specific difference is likely the reason for the lack of overall significance.

### 4.2. PA Diameter and PA/BSA: Diagnosis of PH and Sex Differences

PH seems to be a disease with a predominance in the female sex [19]. As of now, it appears that there is a lack of research investigating the sex differences in the diagnostic methods currently established for PH. In this work, we found that there seems to be small differences between female and male patients when using the PA diameter as a method for PH diagnosis. AUROC analyses for the PA diameters for all investigated sPAP values (40–45–50 mmHg) resulted in highly significant cut-off values for males (cut-off = 29.5 mm; *p* < 0.001). Although we found significant PA diameter cut-off points for sPAP ≥ 40 mmHg (cut-off = 29.5 mm; *p* = 0.044) and sPAP ≥ 50 mmHg (cut-off = 28.5 mm; *p* = 0.029) in female patients, one was not found for sPAP ≥ 45 mmHg (*p* = 0.078). When looking at the PA/BSA sex differences, similar outcomes can be reported. The PA/BSA cut-off values were highly significant for all investigated sPAP values (*p* < 0.001). In females, the PA/BSA cut-off for sPAP ≥ 45 mmHg was, again, not significant. When comparing the AUROC results of males and females in terms of the PA/BSA value, it is evident that the PA/BSA ratio showed a higher diagnostic value for males (highest YI = 0.54) than for females (highest YI = 0.27). The question therefore arises as to why the diagnostic value of the PA/BSA ratio is better in males than in females with regard to the detection of PH. Comparative data are currently lacking to provide an adequate explanation. One potential cause may be attributed to sex differences in ventricular remodeling described in more detail in the following paragraph.

### 4.3. PA Diameter and PA/BSA: Prognosis of Mortality after TAVR and Sex Differences

The severity of PH and concomitant right ventricular dysfunction appears to be higher in males compared with their female counterparts [20,21]. When looking at the PA diameter and PA/BSA ratio, both cut-off values did not seem to be predictive of 1–5 year mortality in females, while the PA diameter and PA/BSA cut-offs in males were both predictive for at least one of the investigated follow-up timepoints. When looking at the Kaplan–Meier calculations for the PA diameter, they seem relevant at first glance, as the PA diameter values in the overall cohort suggest that it can predict 1 year mortality. However, upon closer investigation, this is only true for male patients. Regarding the PA/BSA value, the opposite is true. Survival analysis of the overall cohort showed that the PA/BSA ratio does not predict mortality at any timepoint. This aligns with what we found in our previous work [8]. Yet, when looking at only the male patients, the predictive value of the PA/BSA ratio can be observed. Although the cut-off values for both the PA diameter and PA/BSA ratio differ slightly between males and females, the radiological parameters appear to offer a better risk estimate with respect to long-term survival in the male sex. Presumably, the sex-differential adaptive processes of pathophysiological ventricular remodeling that occur in the setting of severe AS may be responsible for survival differences. Increased pressure and eventual volume loading of the left heart by the AS are subsequently transmitted to the pulmonary circulation and to the right ventricle. Post-capillary PH due to valvular cardiomyopathy generally appears to trigger more rapid negative remodeling in the male heart, leading to poorer adaptation pressure and volume loads, particularly in the right ventricle [21,22]. Progressive deterioration or dysfunction of right ventricular function is ultimately associated with a significantly increased risk of cardiovascular events and thus premature death after TAVR [23]. This fact could provide a potential explanation for why survival after TAVR can be better estimated with echocardiographic evidence of PH in the setting of severe AS, based on the PA diameter or PA/BSA ratio in the male sex, although dilatation of the pulmonary arteries occurs to almost the same extent in both sexes. While our investigations emphasize the diagnostic and prognostic value of CTA measurements between males and females, lifestyle factors like regular exercise and following diets like the Mediterranean diet are also crucial for cardiovascular health, potentially affecting cardiovascular morbidity and mortality [24].

## 5. Conclusions

This study suggests that the CTA-obtained PA diameter and PA/BSA ratio are both useful in the diagnosis of PH and mortality risk stratification in patients with severe AS undergoing TAVR, especially for males. Male patients with PA diameters ≥29.5 mm or PA/BSA values ≥15.7 mm/m^2^ seem to be at a higher risk of death during follow-up after undergoing TAVR.

## 6. Limitations

One major limitation of this study is its retrospective design, decreasing its informative value. To confirm the results of this work, a prospective analysis may be necessary. Additionally, invasive right-heart catheterization to differentiate pre-capillary vs. post-capillary pre-TAVR evaluation of PH is no longer routinely performed in either of the medical centers where the data were obtained. Therefore, we cannot confirm that this patient cohort only included solely left heart-related post-capillary PH.

## Figures and Tables

**Table 1:** Right atrial pressure estimation.

Diameter of IVC	Caliber Fluctuation during Respiration	RAP
≥21 mm	<50%	15 mmHg
No corresponding constellation		8 mmHg
<21 mm	≥50%	3 mmHg

IVC = inferior vena cava; RAP = right atrial pressure.

**Table 2:** Baseline characteristics of the study cohort.

	Total	Men	Women	*p* Value
**Number (%)**				
Total	271 (100.0)	139 (51.3)	132 (48.7)	0.889
Age 60–69 70–79 80–89 ≥90	6/271 (2.2) 63/271 (23.2) 186/271 (68.6) 16/271 (5.9)	5/139 (3.6) 32/139 (23.0) 93/139 (66.9) 9/139 (6.5)	1/132 (0.8) 31/132 (23.5) 93/132 (70.5) 7/132 (5.3)	0.112 0.928 0.529 0.682
BMI <18.5 18.5–24.9 25.0–29.9 30.0–34.9 35.0–39.9 ≥40.0	3/260 (1.2) 119/260 (45.8) 86/260 (33.1) 38/260 (14.6) 13/260 (5.0) 1/260 (0.4)	2/136 (1.5) 60/136 (44.1) 55/136 (40.4) 14/136 (10.3) 5/136 (3.7) 0/136 (0.0)	1/124 (0.8) 59/124 (44.7) 31/124 (23.5) 24/124 (18.2) 8/124 (6.1) 1/124 (0.8)	0.616 0.576 0.008 0.039 0.305 0.294
NYHA ≥ III	142/271 (52.3)	64/139 (46.3)	76/132 (57.6)	0.137
Diabetes mellitus	64/271 (23.6)	31/139 (22.3)	33/132 (25.0)	0.601
Arterial hypertension	219/271 (80.8)	108/139 (77.7)	111/132 (84.1)	0.182
CVD	190/271 (70.1)	103/139 (74.1)	87/132 (65.9)	0.196
Previous myocardial infarction	13/271 (4.8)	4/139 (2.9)	9/132 (6.8)	0.126
Atrial fibrillation	91/271 (33.6)	57/139 (41.0)	34/132 (25.8)	0.008
Previous cardiac surgery	14/271 (5.2)	9/139 (6.5)	5/132 (3.8)	0.318
Pacemaker (before TAVR)	15/271 (5.5)	10/139 (7.2)	5/132 (3.8)	0.220
Malignancy	53/271 (19.6)	33/139 (23.7)	20/132 (15.2)	0.075
Stroke (before TAVR)	18/271 (6.6)	12/139 (8.6)	6/132 (4.5)	0.167
PAOD	15/271 (5.5)	6/139 (4.3)	9/132 (6.8)	0.368
COPD	38/271 (14.0)	23/139 (16.5)	15/132 (11.4)	0.219
LVEF ≤30 31–54 ≥55	13/271 (4.8) 63/271 (23.2) 195/271 (72.0)	8/139 (5.8) 36/139 (25.9) 95/139 (68.3)	5/132 (3.8) 27/132 (20.5) 100/132 (75.7)	0.294 0.265 0.124
sPAP ≥40 ≥45 ≥50 ≥60	120/271 (44.3) 92/271 (34.0) 60/271 (22.1) 26/271 (9.6)	64/139 (46.0) 48/139 (34.5) 31/139 (22.3) 16/139 (11.5)	56/132 (42.4) 44/132 (33.3) 29/132 (22.0) 10/132 (7.6)	0.549 0.835 0.947 0.272
AVR ≥ II°	43/271 (15.9)	18/139 (12.9)	25/132 (18.9)	0.251
MVR ≥ II°	64/271 (23.6)	29/139 (20.9)	35/132 (26.5)	0.271
TVR ≥ II°	44/271 (16.2)	20/139 (14.4)	24/132 (18.2)	0.408
Pacemaker (after TAVR)	62/271 (22.9)	42/139 (30.2)	20/132 (15.2)	0.003
Vascular complications	20/271 (7.4)	11/139 (7.9)	9/132 (6.8)	0.730
Stroke (after TAVR)	9/271 (3.3)	4/139 (2.9)	5/132 (3.8)	0.676
PA ≥ 29.5 mm	127/271 (46.9)	68/139 (48.9)	59/132 (44.7)	0.486
PA/BSA ≥ 15.7 mm/m^2^	144/260 (55.4)	58/136 (42.6)	86/124 (69.4)	< 0.001
**Mean ± SD**				
Age (years)	82.6 ± 4.8	82.7 ± 5.3	82.6 ± 4.4	0.834
Height (cm)	166.9 ± 8.9	172.8 ± 6.6	160.4 ± 6.0	< 0.001
Weight (kg)	73.6 ± 14.7	77.7 ± 12.9	69.3 ± 15.2	< 0.001
BMI (kg/m^2^)	26.4 ± 5.1	26.0 ± 4.0	26.9 ± 6.0	0.170
LVEF (%)	55.3 ± 10.1	54.1 ± 11.0	56.5 ± 8.9	0.061
LVEDD (mm)	44.1 ± 17.4	45.3 ± 18.7	42.6 ± 15.9	0.351
IVSd (mm)	14.7 ± 2.9	15.0 ± 3.2	14.3 ± 2.4	0.090
AV Vmax (m/s)	4.3 ± 0.9	4.4 ± 1.1	4.3 ± 0.8	0.226
AV dpmax (mmHg)	78.3 ± 19.2	79.3 ± 18.2	77.3 ± 20.1	0.411
AV dpmean (mmHg)	47.9 ± 19.2	48.0 ± 11.5	47.7 ± 12.6	0.796
TAPSE (mm)	22.4 ± 4.4	22.6 ± 4.6	22.1 ± 4.2	0.554
sPAP (mmHg)	35.9 ± 18.2	36.9 ± 18.0	34.8 ± 18.5	0.338
PA (mm)	29.3 ± 5.1	29.4 ± 5.3	29.1 ± 4.9	0.552
PA/BSA	16.2 ± 3.0	15.4 ± 2.7	17.0 ± 3.0	< 0.001
**Median ± IQR**				
STS score	2.4 ± 1.7	2.0 ± 1.2	3.5 ± 2.6	< 0.001
Creatinine (mg/dL)	1.0 ± 0.5	1.0 ± 0.5	0.9 ± 0.4	0.005
BNP (pg/mL)	1195.0 ± 2258.6	1098.0 ± 2921.5	1228.0 ± 1941.6	0.148
HK (%)	39.0 ± 7.0	40.3 ± 7.4	38.7 ± 6.0	0.001
HB (g/dL)	13.1 ± 2.3	13.6 ± 2.6	12.9 ± 1.6	< 0.001
CK (U/L)	80.0 ± 84.5	80.0 ± 102.5	82.0 ± 64.8	0.130

BMI = body mass index; NYHA = New York Heart Association; CVD = coronary vascular disease; TAVR = transcatheter aortic valve replacement; PAOD = peripheral artery occlusive disease; COPD = chronic obstructive pulmonary disease; LVEF = left ventricular ejection fraction; sPAP = systolic pulmonary artery pressure; AVR = aortic valve regurgitation; MVR = mitral valve regurgitation; TVR = tricuspid valve regurgitation; PA = pulmonary artery; BSA = body surface area; SD = standard deviation; LVEDD = left ventricular end diastolic diameter; IVSd = interventricular septum diameter; TAPSE = tricuspid annular plane systolic excursion; IQR = interquartile range; STS score = The Society of Thoracic Surgeons score; BNP = brain.

**Table 3:** Univariate and multivariate cox regression analysis detecting 1, 3, and 5 year mortality in male patients.

Cox Regression Analysis	Univariate		Multivariate	
	Hazard Ratio (95% CI)	*p* Value	Hazard Ratio (95% CI)	*p* Value
1 year mortality male				
Stroke after TAVR	3.522 (0.832–14.919)	0.087	14.287 (2.733–74.691)	0.002
sPAP	2.041 (1.410–2.953)	<0.001	2.023 (1.245–3.289)	0.004
PA	1.705 (1.181–2.463)	0.004	1.482 (0.981–2.240)	0.062
PA/BSA	1.682 (1.100–2.570)	0.016	0.546 (0.210–1.418)	0.214
3 year mortality male				
BNP	1.227 (1.001–1.504)	0.049	1.045 (0.633–1.723)	0.864
HB	0.782 (0.583–1.048)	0.099	0.858 (0.544–1.354)	0.512
Troponin	1.447 (0.963–2.174)	0.075	0.900 (0.397–2.042)	0.801
sPAP	1.871 (1.392–2.516)	<0.001	1.772 (1.070–2.933)	0.026
PA	1.669 (1.236–2.253)	0.001	1.570 (0.997–2.473)	0.052
PA/BSA	1.748 (1.250–2.442)	0.001	0.727 (0.283–1.866)	0.508
5 year mortality male				
Age	1.257 (0.980–1.612)	0.072	1.087 (0.622–1.897)	0.770
STS score	1.473 (0.991–2.190)	0.055	1.256 (0.601–2.625)	0.544
BNP	1.233 (1.027–1.482)	0.025	1.020 (0.604–1.722)	0.941
HK	0.782 (0.609–1.004)	0.054	1.474 (0.444–4.891)	0.526
HB	0.791 (0.619–1.010)	0.060	0.810 (0.549–1.195)	0.289
Troponin	1.361 (0.955–1.938)	0.088	0.363 (0.080–1.639)	0.188
sPAP	1.612 (1.258–2.065)	<0.001	1.371 (0.730–2.578)	0.327
PA	1.449 (1.118–1.878)	0.005	1.655 (1.110–2.469)	0.014
PA/BSA	1.639 (1.230–2.184)	0.001	0.453 (0.137–1.494)	0.193

sPAP = systolic pulmonary artery pressure; PA = pulmonary artery; BSA = body.

**Table 4:** Univariate and multivariate cox regression analysis detecting 1, 3, and 5 year mortality in female patients.

Cox Regression Analysis	Univariate		Multivariate	
	Hazard Ratio (95% CI)	*p* Value	Hazard Ratio (95% CI)	*p* Value
1 year mortality female				
HK	0.564 (0.312–1.019)	0.058	0.621 (0.317–1.217)	0.165
HB	0.565 (0.309–1.034)	0.064	1.643 (0.162–16.628)	0.674
CK	1.243 (1.087–1.420)	0.001	1.243 (1.087–1.420)	0.001
Pacemaker after TAVR	3.350 (0.980–11.449)	0.054	3.134 (0.779–12.611)	0.108
3 year mortality female				
STS score	1.654 (0.922–2.966)	0.091	1.507 (0.716–3.169)	0.280
CK	1.243 (1.089–1.420)	0.001	12.169 (1.020–145.170)	0.048
LVEF	0.694 (0.461–1.045)	0.080	0.622 (0.229–1.690)	0.352
5 year mortality female				
CK	1.240 (1.084–1.418)	0.002	1.269 (1.101–1.463)	0.001
IVSd	1.334 (0.967–1.840)	0.079	1.351 (0.961–1.899)	0.083
Stroke after TAVR	4.805 (1.891–12.209)	0.001	6.751 (2.327–19.591)	0.000

HK = hematocrit; HB = hemoglobin; CK = creatinine kinase; LVEF = left ventricular ejection fraction; IVSd = interventricular septum diameter.

## Data Availability

The data presented in this study are available on request from the corresponding authors.
